# Aedes (Stegomyia) albopictus (Skuse), a potential new Dengue vector in southern Cameroon.

**DOI:** 10.3201/eid0706.010631

**Published:** 2001

**Authors:** D Fontenille, J C Toto

**Affiliations:** Organisation de Coordination pour la Lutte contre les Endémies en Afrique Centrale, Yaoundé, Cameroon. didier.fontenille@mpl.ird.fr

## Abstract

Aedes albopictus, a mosquito vector of Dengue virus, has been recorded for the first time in Cameroon. Entomologic surveys in 2000 demonstrated that it is widespread in southern Cameroon, colonizing a wide variety of breeding sites and biting humans in every district surveyed. The presence of this vector increases the risk for emergence of dengue in Cameroon.


### *Aedes* (*Stegomyia*) *albopictus* (Skuse), a Potential New Dengue Vector in Southern Cameroon

**Didier Fontenille*† and Jean Claude Toto***
*Organisation de Coordination pour la Lutte contre les Endémies en Afrique Centrale (OCEAC), Yaoundé, Cameroon; and †Institut de Recherche pour le Développement, Montpellier, France

*Aedes albopictus*, a mosquito vector of *Dengue virus*, has been recorded for the first time in Cameroon. Entomologic surveys in 2000 demonstrated that it is widespread in southern Cameroon, colonizing a wide variety of breeding sites and biting humans in every district surveyed. The presence of this vector increases the risk for emergence of dengue in Cameroon.

*Aedes albopictus* is among the most important arbovirus vectors in the world, particularly for *Dengue virus* (DV) (1). The microhabitats of its larvae are mainly tree holes and a wide variety of containers. The eggs can survive desiccation for several months. The adult biology of *Ae. albopictus* is similar to that of the urban population of *Ae. aegypti*, a dengue and yellow fever vector (2). The characteristics of its eggs, its close association with humans, and increasing intercontinental travel have favored the expanding global distribution of this Asian species (3).

*Ae. albopictus* was recorded in North America as early as 1972. Established populations were detected in 1985, imported from Asia in used tires (4). Its presence was reported in Brazil in 1986, then in the Pacific islands and the Caribbean islands, and more recently in Europe (Albania, Italy, and France) (5,6).

In Africa, this vector was observed for the first time in 1989 in South Africa. After its eggs were introduced in tires from Japan (7), *Ae. albopictus* was recorded in Nigeria in 1991 (8), where it has become widespread. To date, this vector has not been observed in other sub-Saharan countries.

Surveys of *Ae. aegypti* distribution conducted from 1950 to 1995 in several regions in Cameroon did not record *Ae.albopictus*. A large trial conducted in 1976 in 84 locations recorded 1,112 *Ae. aegypti*-positive larval development sites but none positive for *Ae. albopictus* (9). Moreover, entomologic investigations during two yellow fever epidemics in 1990 and 1995 in North Cameroon recorded only *Ae. aegypti* (10; unpub. report: Enquête entomo-épidémiologique sur deux cas mortels de fièvre jaune survenus dans la ville de Ngaoundéré [Province de l'Adamaoua, Cameroun], ORSTOM laboratory, Centre Pasteur of Cameroon, 1995). DV has never been isolated in Cameroon.

In October 1999, one of the authors captured biting *Ae.albopictus* females, which prompted a thorough investigation to monitor the presence, distribution, and biology of this species in southern Cameroon.

Address for correspondence: Didier Fontenille, Laboratoire de Lutte contre les Insectes Nuisibles, Institute de Recherche pour le Développement, 911 av. Agropolis, BP 5045, 34032 Montpellier Cedex 1, France; fax: 33-04-6754-2044; e-mail: didier.fontenille@mpl.ird.fr

### Materials and Methods

### Study Sites

Surveys were conducted in the two main cities of Cameroon: Douala, pop. 1,400,000 (4°00'N, 9°45'E), commercial harbor and largest city in Cameroon, and Yaoundé, pop. 1,300,000 (3°4150'N, 11°30'E) the capital city, located at an altitude of 800 m. Entomologic studies were also conducted in Campo (2°30'N, 9°50'E; pop. 4,000), Edea (3°45'N, 10°10'E; pop. 100,000), and Bafia (4°45'N, 11°15'E; pop. 50,000).

### Larvae and Adult Mosquito Collections

Larval development sites of mosquitoes were investigated in four districts in Yaoundé (Gare, Cité Verte, Brasseries, and Biyemassi), four districts in Douala (Dibom, New Bell, Bonaberi, and Makepe), and three districts in Edea and Bafia. Approximately 20 potential breeding sites containing water were sampled in each district in Yaoundé and Douala; an average of seven breeding sites were sampled in each district in Edea and Bafia. A breeding site was recorded as positive when it contained mosquito larvae or pupae, whatever the species.

Biting behavior of mosquitoes was checked by five adult volunteers in the districts of Yaoundé, Douala, and Campo. These volunteers collected mosquitoes landing on their arms or legs from 5:00 to 6:30 p.m. All surveys were conducted in October and November 2000, at the end of the long rainy season.

Larvae and adults were identified by the morphologic identification keys and morphologic descriptions of African *Aedes* species (11-13). Male genitalia were dissected and examined under a microscope.

### Results

*Ae. albopictus* was present in all five towns and in every district sampled. Species identification was confirmed on larvae and adult males and females. Of the positive larval development sites sampled, 75% of 36 in Yaoundé and 45% of 53 in Douala contained *Ae. albopictus* larvae. *Ae. albopictus* was found in five breeding sites in Edea and seven in Bafia (Table).

The volume of water in *Ae. albopictus*-positive breeding sites ranged from 50 mL to 100 L. Species found together in

Table. Breeding sites found positive for *Aedes albopictus* in 2000, southern Cameroon

| Types of breeding sites containing water | Number of positive / sites sampled of each type | Percent Positive (%) |
|---|---|---|
| Used tire | 36/77 | 47 |
| Plastic container | 7/27 | 26 |
| Can and broken bottle | 9/30 | 30 |
| Plastic cup | 3/6 | 50 |
| 200-L barrel | 0/7 | 0 |
| Abandoned car part | 6/35 | 17 |
| Cement washtub | 0/4 | 0 |
| Flowerpot | 0/2 | 0 |
| Tree hole | 0/4 | 0 |
| Cow horn | 0/4 | 0 |
| Cocoa pod | 0/4 | 0 |
| Enameled plate | 1/6 | 17 |
| Snail shell | 1/3 | 33 |

the same sites were *Ae. aegypti*, *Anopheles gambiae* s.s., *Culex* gr. *decens*, *Cx. quinquefasciatus*, *Cx. poicilipes*, *Cx. duttoni*, *Cx.* (*Culiciomyia*) sp., *Cx.* (*Lutzia*) *tigripes*, and *Eretmapodites quinquevittatus*. Of breeding sites positive for *Ae. albopictus* or *Ae. aegypti*, both species were found together in 68% of sites in Yaoundé, 50% in Douala, 33% in Edea, and 38% in Bafia.

Late afternoon captures of adults demonstrated that *Ae.albopictus* is anthropophilic. The average number of *Ae.albopictus* females collected per volunteer from 5:00 to 6:30 p.m. was 1.1 (range 0 to 8) in Douala and 3.0 (range 0 to 17) in Yaoundé. Other species collected were *Ae. aegypti*, *An. gambiae* s.s., *Cx. quinquefasciatus*, *Cx. antennatus*, *Cx. perfuscus*, *Cx.* from *neavei* group, *Cx.* from *decens* group, *Er. quinquevittatus*, *Mansonia uniformis*, and *Ma. africana*. *Ae. albopictus* was the species most often captured, accounting for 35% of all the mosquitoes.

### Conclusions

In 2000, *Ae. albopictus* was already widespread in South Cameroon. It was present in all the districts and towns sampled, in a wide variety of breeding sites, the most common being used tires, as described elsewhere (2). Used or retread tires are imported regularly from the United States, Nigeria, and South Africa, countries where *Ae. albopictus* is present (unpub. data, Ministry of Commerce, Cameroon). This observation strongly suggests that this species was introduced to Cameroon in this way, likely on multiple occasions in different regions.

The species is frequently associated with *Ae. aegypti*, as observed in other countries (14). Some observations from regions where *Ae. albopictus* was recently introduced suggest it tends to supplant *Ae. aegypti* (15). Such interspecific competition was experimentally observed in an insectary (16). The absence of *Ae. albopictus* in the lists of mosquito species observed in Cameroon before 1995 suggests that this species has colonized South Cameroon recently and that its diffusion has been rapid, as was the case in neighboring Nigeria and in America and Europe.

*Ae. albopictus* is a competent vector for DV. Because this disease is expanding in the world (17), data are needed on the actual distribution of *Ae. albopictus* throughout Cameroon and the potential risk for transmission of arbovirus. Surveillance of used tires, which seem to be its preferred breeding sites, can provide maximum information on species distribution at the lowest cost-effective rate. The presence of this vector, in association with *Ae. aegypti*, increases the risk for emergence of dengue in Cameroon.

### Acknowledgments

We thank Sue Gartland for help with the English translation.

This work was funded by l'Institut de Recherche pour le Développement (IRD), France.

Dr. Fontenille is head of the medical entomology laboratory of OCEAC (Organisation de Coordination pour la lutte contre les Endémies en Afrique Centrale), Yaounde, Cameroon. For 20 years, he has conducted research in Africa on arboviruses and malaria vectors.

Mr. Toto is a technician in the medical entomology laboratory of OCEAC.

### References

1. Shroyer DA. *Aedes albopictus* and arboviruses: a concise review of the literature. J Am Mosq Control Assoc 1987;2:424-8.
2. Hawley WA. The biology of *Aedes albopictus*. J Am Mosq Control Assoc 1988;4:1-40.
3. Rodhain F. Problèmes posés par l'expansion d'*Aedes albopictus*. Bulletin de la Société de Pathologie Exotique 1996;89:137-41.
4. Hawley WA, Reiter P, Coperland RS, Pumpuni CB, Craig GB Jr. *Aedes albopictus* in North America: probable introduction in used tires from Northern Asia. Science 1987;236:1114-6.
5. Dalla Pozza G, Majori G. First record of *Aedes albopictus* establishment in Italy. J Am Mosq Control Assoc 1992;8:318-20.
6. Schaffner F, Karch S. Première observation d'*Aedes albopictus* (Skuse, 1894) en France métropolitaine. Comptes Rendus de l'Académie des Sciences III 2000;323:373-5.
7. Cornel AJ, Hunt RH. *Aedes albopictus* in Africa? First records of live specimens in imported tires in Cape Town. J Am Mosq Control Assoc 1991;7:107-8.
8. Savage HM, Ezike VI, Nwankwo ACN, Spiegel R, Miller BR. First record of breeding populations of *Aedes albopictus* in continental Africa: implications for arboviral transmission. J Am Mosq Control Assoc 1992;8:101-3.
9. Rickenbach A, Button JP. Enquête sur les vecteurs potentiels domestiques de fièvre jaune au Cameroun. Cahiers ORSTOM, série Entomologie médicale et Parasitologie 1977;15:93-103.
10. Vicens R, Robert V, Pignon D, Zeller H, Digoutte JP. L'épidémie de fièvre jaune du Nord Cameroun en 1990: premier isolement du virus amaril au Cameroun. Bull World Health Organ 1993;71:173-6.
11. Edwards FW. Mosquitoes of the Ethiopian region. III. Culicine adults and pupae. London: British Museum Natural History; 1941. p. 499.
12. Hopkins GHE. Mosquitoes of the Ethiopian region. I. Larval bionomics of mosquitoes and taxonomy of *Culicine larvae*. 2nd ed. London: British Museum Natural History; 1952. p. 355.
13. Jupp PG. Mosquitoes of Southern Africa. Hartebeespoort (South Africa): Ekogilde Publishers; 1996. p. 156.
14. Chan KL, Chan YC, Ho BC. *Aedes aegypti* (L.) and *Aedes albopictus* (Skuse) in Singapore city. Competition between species. Bull World Health Organ 1971;44:643-9.
15. Hobbs JH, Hughes EA, Eichold BH II. Replacement of *Aedes aegypti* by *Aedes albopictus* in Mobile, Alabama. J Am Mosq Control Assoc 1991;7:488-99.
16. Barrera R. Competition and resistance to starvation in larvae of container-inhabiting *Aedes* mosquitoes. Ecol Entomol 1996;21:117-27.
17. Gubler DJ. Dengue and dengue hemorrhagic fever. Clin Microbiol Rev 1998;11:480-96.
